# Eye-tracking metrics for estimating workload and characterizing errors in conflict detection and resolution during simulated en route air-traffic control

**DOI:** 10.3389/fpsyg.2025.1644721

**Published:** 2025-12-10

**Authors:** José A. Navia, Jorge Ibáñez-Gijón, David Travieso, Aitor Montes, Patricia López de Frutos

**Affiliations:** 1 Dpto. Psicología Básica, Facultad de Psicología, Universidad Autónoma de Madrid, Madrid, Spain; 2 Dpto. Ciencias de la Educación, Facultad de Educación, Universidad de Alcalá, Guadalajara, Spain; 3 CRIDA A.I.E. ATM R&D + Innovation Reference Centre, Madrid, Spain

**Keywords:** air-traffic control, eye tracking, mental workload, conflict detection, conflict resolution, human factors

## Abstract

Growing traffic density and airspace complexity demand adaptive decision-support tools that anticipate when controllers are approaching overload or conflicts are mishandled. Ocular behavior offers a single, unobtrusive stream that simultaneously reflects global mental workload (MWL) and moment-to-moment attentional allocation. The present study examined whether eye-tracking metrics can estimate MWL and expose the mechanisms underlying errors in conflict detection and resolution during simulated en route control. Twenty-four novice participants worked six 16-min radar scenarios that varied traffic load and sector complexity. A remote eye-tracker recorded pupil diameter, blink dynamics, and fixations on static and aircraft-centered areas of interest, while subjective MWL was sampled with the Instantaneous Self-Assessment and NASA-TLX scales. Higher traffic density increased self-reported MWL, enlarged pupils, reduced blinks and blink durations, and concentrated fixations inside the active sector, whereas higher traffic complexity increased MWL, reduced blinks, and concentrated fixations inside the active sector. Blink rate and pupil size accounted for most of the variance in MWL (up to 94%). In addition, two scripted conflict events were examined in greater detail. In the simpler conflict, errors primarily stemmed from failures in detection. Successful resolutions were characterized by sustained gaze on both converging aircraft and a higher frequency of altitude-change clearances, while failures showed reduced fixation times and a lack of interventions. In contrast, errors in the more complex conflict resulted from planning breakdowns despite initial detection. Successful resolutions in this case typically involved at least two interventions, whereas failures were associated with prolonged fixation times but insufficient corrective action. Thus, global ocular indices provide precise estimates of MWL, and gaze-action couplings can help anticipate errors in conflict detection and resolution. Embedding both levels of inference in adaptive ATC support systems could enable real-time MWL management, and proactive mitigation of separation-loss events.

## 1 Introduction

Air traffic control (ATC) plays a decisive role in the safety and efficiency of air transport. En route air-traffic conflicts (ATCOs) are responsible for monitoring aircraft that are already airborne and cruising, ensuring they remain on their planned trajectories and intervening when necessary to prevent loss-of-separation conflicts. Because any lapse can have severe consequences, it is essential to keep ATCOs’ mental workload (MWL) within acceptable limits. Achieving this requires a detailed understanding of how task-specific demands contribute to MWL (Young et al., 2015), which in turn calls for robust methods to quantify and interpret MWL in operationally relevant settings (Loft et al., 2007; Pagnotta et al., 2021).

Three broad classes of metrics have been used to characterize MWL in ATC: performance outcomes, subjective self-reports, and behavioral or psychophysiological indices (Hilburn, 2004). Performance-based metrics can be misleading because ATCOs actively compensate for rising task difficulty to preserve performance levels (Sperandio, 1971). Subjective instruments such as the NASA Task Load Index (NASA-TLX; Hart, 2006; Hart and Staveland, 1988) and the Instantaneous Self-Assessment (ISA; Jordan and Brennen, 1992) are sensitive to traffic load (Ibáñez-Gijón et al., 2023; Loft et al., 2007) but suffer from discontinuity, recall bias, and large inter-judge variability (Ahlstrom and Friedman-Berg, 2006; Aricò et al., 2019). In addition, subjective measures cannot be administered covertly in live operations. By contrast, psychophysiological indicators such as eye-tracking, heart-rate variability, or electrodermal activity, offer continuous, unobtrusive sampling and thus increasingly represent the preferred method for monitoring ATCO MWL in both research and operational environments.

Among the psychophysiological measures, global eye-tracking metrics such as pupil size or blink rate have demonstrated considerable success in estimating average MWL during an ATC session. Task demands show consistent correlations with various ocular parameters including pupil diameter (Bernhardt et al., 2019; Rodríguez et al., 2015), blink rate (Brookings et al., 1996), blink duration (Ahlstrom and Friedman-Berg, 2006), and closing blink speed (Meyer et al., 2022), although these relationships exhibit variability across conditions and individuals (Kuo et al., 2017; Tautz and Tenoort, 2001; Vogt et al., 2006). Session-level MWL indices are valuable for airspace planning, but they cannot help mitigate the moment-to-moment accident risk inherent in ATC operations. For that, continuous tracking and regulation of ATCOs’ MWL is needed.

Task-evoked changes in global ocular metrics offer particular promise for real-time MWL analysis due to their rapid onset (100–200 ms) and sensitivity to within-task, between-task, and between-individual variations (Beatty, 1982). Ahlstrom and Friedman-Berg (2006) demonstrated linear relationships between traffic density and ocular metrics in high-fidelity weather simulations: Pupil diameter increased by 0.012 mm per aircraft from baseline, while blink duration decreased by 4 ms per aircraft in the airspace. Multiple-regression and neural-network models based solely on these metrics explained 60–84% of minute-by-minute traffic load variance, reaching 92% accuracy in high-load conditions. Recent advances have further improved classification performance, with Lemetti et al. (2024) achieving overall accuracies above 95% and precisions above 84% for MWL using multiple ocular metrics.

Notwithstanding these achievements in transient MWL estimation, global ocular metrics provide limited information regarding the allocation of attention within traffic scenarios (Martin et al., 2011). Consequently, these metrics cannot determine which specific aircraft captured the ATCOs’ attention during loss-of-separation events or explain the underlying causes of mishandled incidents (Hunter and Parush, 2009). Understanding delayed or missed conflict detection and resolution (CD&R) requires a more detailed analysis that links fixation patterns on the aircraft pairs involved in conflicts with the ATCOs’ tactical responses.

Initial attempts to address this question employed simplified experimental displays featuring single converging aircraft pairs. Under these controlled conditions, extended dwell times on converging aircraft and frequent transitions between them and the conflict site predicted successful CD&R, while dispersed scan patterns preceded failures (Hunter and Parush, 2009). When research progressed to realistic multi-target simulations, scanpaths became considerably more complex and variable. Kang and Landry (2014) developed a dynamic clustering algorithm that assigned fixations to the nearest aircraft and subsequently presented expert scan patterns to novice ATCOs, resulting in approximately 70% reduction in false-alarm rates. Further research proposed a methodology to categorize ATCOs’ scanpaths into simpler canonical patterns (circular, spiral, linear) and demonstrated that increasing traffic density can induce a transition from broad circular scanning to narrower linear patterns (McClung and Kang, 2016). Palma Fraga et al. (2021) used a similar approach to simplify scanpaths and map these patterns to the search and resolution heuristics obtained from expert ATCOs interviews. Participants employed systematic search patterns (circular, spiral, linear, or quadrant-based) to extract operationally relevant information, applied a cognitive information hierarchy (altitude > direction > speed), and generally preferred altitude or direction changes over speed adjustments when mitigating imminent potential conflicts.

Recent investigations have adopted the Conflict Life-Cycle model to integrate visual metrics within a task-relevant analytical framework (Meyer et al., 2022; see also Gronlund et al., 2001; Pawlak et al., 1996). This model segments CD&R into four phases: detection, planning (solution probing), implementation, and monitoring phases. Meyer et al. (2022) investigated how en route ATCOs use decision cues. Using a retrospective think-aloud method with 13 ATCOs presented with videos of their scanpaths during the resolution of a simple simulated conflict, they identified and categorized these cues across the three phases of the model (the implementation phase was not considered), revealing significant individual differences in how information is processed and decisions are made. The planning step emerged as the most complex, highlighting the need for future automation to align with ATCOs’ decision-making logic and timing. These results confirmed the possibility to use eye-tracking as an indicator of phase-specific information pickup. In a follow-up study, Nordman et al. (2023) combined high-resolution gaze data with ATCOs input logs and applied topic modeling (an unsupervised machine learning technique) to infer Life-Cycle phases in real time. These automatically learned strategies were validated with the phase-specific cues obtained by Meyer et al. (2022). However, despite its remarkable performance in categorizing the phases of CD&R, this approach requires the integration of massive amounts of data and is not focused on identifying or anticipating the causes of errors in CD&R.

In this study, we propose an intermediate approach to characterize errors in CD&R that seeks a balance between the interpretability of controlled studies and the ecological validity of realistic environments. This method avoids the complexity of full scanpath analysis while still enabling accurate predictions of CD&R performance. While our simulated setup affords only moderate ecological validity relative to real operations, it bridges controlled single-pair studies and opaque full scanpath models by linking dynamic AOI gaze to specific interventions in complex multi-aircraft scenarios. For each conflict, we analyze how ATCO intervention patterns (that is, the count, type -altitude/speed-, and timing of controller clearances within the conflict window) relate to the cumulative fixation time on the involved aircraft(s). To identify the sources of errors, we conduct regression analyses on gaze and intervention measures that differ significantly between solved and unsolved trials. This approach preserves interpretability by linking visual attention to concrete actions in complex scenarios and reduces analytical complexity by focusing on accumulated fixation time within dynamic areas of interest (AOIs). Compared to static AOI methods (Hauland, 2008), this dynamic AOI approach offers a simpler, more direct way to assess whether attention was effectively directed to relevant targets at critical moments. Note, however, that in this study we used both static AOIs (sector, out-of-sector, flight-strip) to characterize effects on global attentional allocation of session level average MWL, and dynamic AOIs that translated with each aircraft to quantify target-specific attention allocation during CD&R.

The present study addresses two questions. First, can global metrics of visual behavior such as pupil size, blink rate and duration, and fixations on static scenario-level AOIs, serve as reliable indicators of MWL on ATC tasks? Second, can intervention patterns and simplified aircraft-specific metrics of gaze behavior, be used to identify and anticipate breakdowns in CD&R?

To answer these questions, participants engaged in a series of simulated en route ATC tasks designed to systematically manipulate MWL. Eye movements and intervention behaviors were continuously recorded across scenarios. Based on prior research, we hypothesized that (1) increased MWL would be associated with larger pupil diameter, lower blink rate and duration, and a shift in fixation patterns (increasing attention to the sector and reducing attention to peripheral AOIs). Furthermore, we hypothesized that (2) gaze dynamics and intervention patterns could be used to infer the underlying source of CD&R errors. We expected that error mechanisms would vary by conflict complexity: Errors in simple conflicts would primarily result from failures in detection, whereas errors in complex conflicts would more often reflect planning failures following successful detection. To test this, we analyzed in detail two scripted conflict events that differed in their structural complexity, duration, and number of aircraft involved.

## 2 Materials and methods

### 2.1 Participants

A power analysis with G*Power (Faul et al., 2007) for a repeated-measures design (one group, six measurements) indicated that 19 participants would be sufficient to achieve 80% power (1-*β* = 0.80, *α* = 0.05) for detecting effect sizes of *η_p_*^2^ = 0.06. Twenty-four university students (*M* age = 19.5 ± 1.3 years) with normal or corrected-to-normal vision and no prior experience in ATC tasks volunteered. All signed informed consent, and the study was approved by the local ethics committee (UAM-CEI-110-2163).

### 2.2 Material

**Figure 1:**
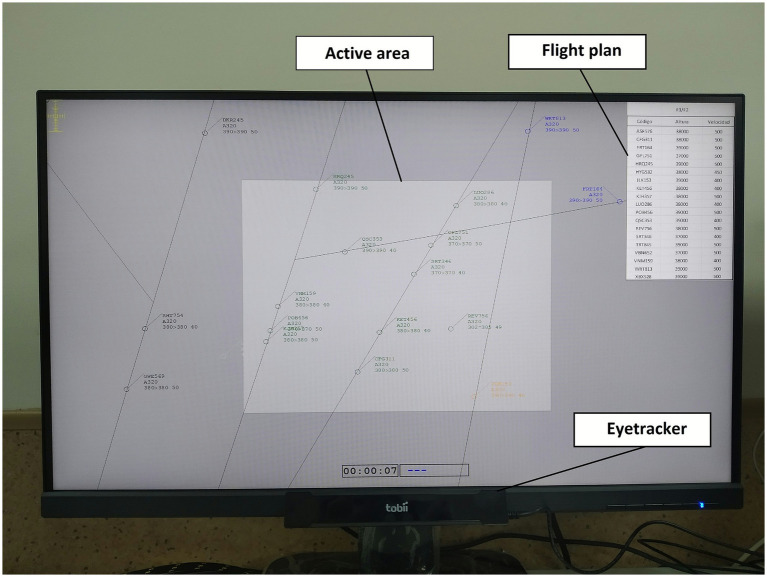
Experimental set up during data collection.

ATC scenarios were presented with the ATC-Lab Advanced simulator (Fothergill et al., 2009) on a 27-inch HD monitor positioned 60 cm from the participant (Figure 1). A standard mouse and keyboard were used as input devices. Participants sat in front of the simulator without a chinrest or any physical constraints on their movements, although the experimenter monitored them to ensure a sufficiently constant head distance. Scenario parameters replicated the specifications of Ibáñez-Gijón et al. (2023). Eye movements were recorded with a Tobii X2 compact eye tracker (30 Hz) and Tobii Studio v3.4.8 (Tobii, Danderyd, Sweden). Subjective MWL was assessed with ISA (Jordan and Brennen, 1992) and NASA-TLX (Hart, 2006; Hart and Staveland, 1988).

### 2.3 Design

Task workload was manipulated in a 2 × 3 within-subjects factorial design: two traffic levels (six vs. twelve simultaneous aircraft) and three airspace-complexity levels (low, medium, and high). Complexity followed the COMETA MWL model, which considers (a) number of standard routes, (b) route crossing points, (c) non-standard routes, (d) flights in evolution (non-zero vertical velocity), and (e) loss-of-separation events (Ibáñez-Gijón et al., 2023, §4.1). The six resulting scenarios were presented in counterbalanced order.

### 2.4 Procedure

Participants first completed a practice session with full instructions that lasted 60 min (Ibáñez-Gijón et al., 2023). They then worked through the six 16-min experimental scenarios. Before each scenario, a nine-point screen calibration secured eye-tracking accuracy using Tobii’s calibration procedure, and seating was adjusted as required. In order to ensure the calibration quality, we continuously tracked the head position of the participants to be within the recommended distance (between 50 and 80 cm). The average head distance of the participants was 59 ± 1 cm across conditions (see Supplementary Material 1 for details and statistical tests). No calibration drift was expected during the 16-min scenario runs due to the continuous control of the head distance, the highly stationary positions of the participants, and the absence of mechanical perturbations during the trial. Testing took place in a windowless room under constant luminance. Participants verbalized each action (e.g., altitude change) and awaited the experimenter’s verbal confirmation to emulate pilot communication. They also reported ISA ratings every 2 min during each scenario. Immediately afterwards, they completed the NASA-TLX. A voluntary 10 min break followed the third scenario, and the whole session lasted around 3 h.

### 2.5 Measures

#### 2.5.1 MWL

ISA ratings range from 1 to 7; the mean of the eight ratings per scenario was analyzed. NASA-TLX component scores range from 1 to 20; their raw sum (range 6–120) was analyzed (Hart, 2006). Both measures have been shown to co-vary with the task workload manipulations used here (Ibáñez-Gijón et al., 2023). We compared them to assess whether the eye-tracking apparatus introduced additional MWL.

#### 2.5.2 Pupil size and blinks

From the Tobii 30 Hz output we extracted mean pupil diameter (mm), mean eye-tracker distance (cm), blink count, and mean blink duration. Blinks were defined as the simultaneous loss of eye data lasting 100–600 ms (Kwon et al., 2013). The mean values of these measures during the 16-min scenarios were submitted to statistical analysis.

#### 2.5.3 Static AOIs fixations

**Figure 2:**
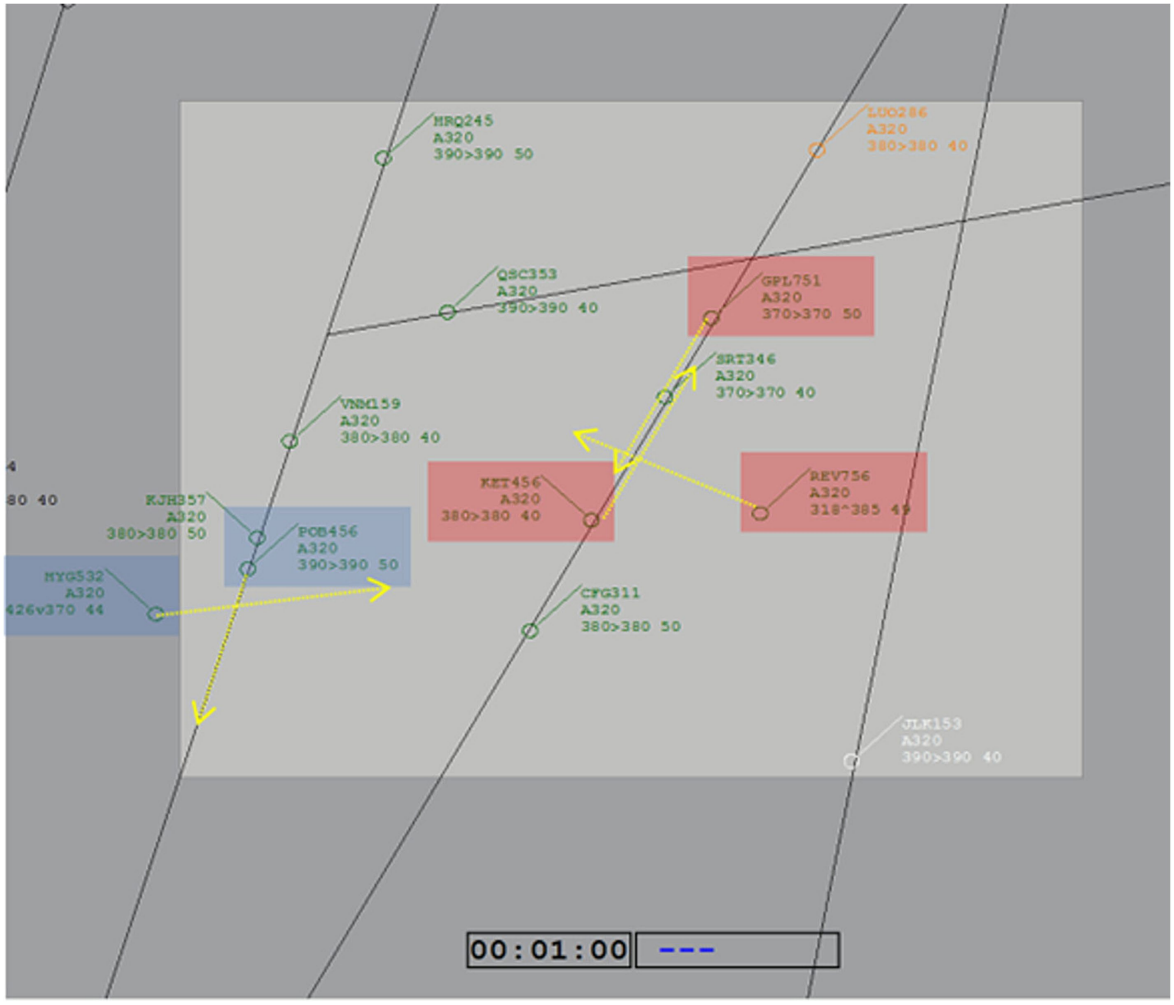
Scenario 12-high depicting the two conflicts analyzed. Red and blue rectangles highlight the flights involved in each of the two conflicts considered in this study. Yellow lines represent the heading of each flight.

Total fixation count and duration for the 16-min scenarios were obtained for three predefined AOIs on the 61 × 34 cm radar display: (a) active sector (central 27.5 × 20.5 cm rectangle, corresponding to a visual angle of 25.81 × 19.39 °), (b) surrounding airspace (out-of-sector), and (c) the flight-plan sheet (17 × 7.5 cm) in the top-right corner (Figure 2). A fixation was any gaze that remained within 1° for at least 100 ms.

#### 2.5.4 CD&R behavior

Detailed gaze behavior and intervention commands were analyzed during two predefined potential conflicts with converging headings that appeared at the start of the 12-high scenario. Figure 2 depicts the two conflicts with a color-coded highlight of the aircraft involved and their respective movement directions (see also Supplementary Material 2 for snapshots of the conflicts evolving in time). The Easy conflict (highlighted by blue rectangles) was programmed to violate safety boundaries 140 s after scenario onset if left unresolved. It involved two aircraft: POB456, following a standard route, and HYG532, climbing along a non-standard trajectory. According to the COMETA model, the calculated complexity of this conflict was 0.20. For reference, COMETA assigns a complexity value of 0.10 to aircraft in stable evolution and 0.15 to those operating on non-standard routes. The Difficult conflict (indicated by the red highlight rectangles) was programmed to violate safety boundaries 240 s after scenario onset and involved three aircraft: GPL751 and KET456, both following standard routes, and REV756, climbing along a non-standard trajectory. Flight SRT346 appeared on a similar trajectory but was not involved in the conflict. The COMETA model estimated the complexity of this conflict at 0.30, indicating a substantially higher demand.

A conflict was considered unsolved if the safe separation boundaries (at least 1,000 ft. vertically and 5 NM laterally) were violated at any point during the scenario. For each conflict, we recorded the following variables: (a) whether safety boundaries were maintained or not; (b) the number of altitude and speed interventions per flight; and (c) the cumulative fixation time on each aircraft (starting from sector entry for HYG532). Dynamic AOIs for each aircraft involved in the conflicts were defined (with the same size for all aircraft) and manually adjusted frame by frame to changes in flag information tags to continuously track their movements as illustrated in Figure 2 and Supplementary Material 2.

### 2.6 Statistical analysis

All dependent variables were submitted to 2 (traffic) × 3 (complexity) repeated-measures ANOVAs. Normality of residuals was checked via *Q-Q* plots and Kolmogorov–Smirnov tests; when sphericity was violated, Huynh-Feldt corrections were applied. Bonferroni-adjusted pairwise comparisons followed significant effects. Spearman’s *ρ* assessed correlations between global eye-tracking indices and subjective MWL.

CD&R data were analyzed with Mann–Whitney *U* tests (“solvers” vs. “non-solvers”). In addition, Spearman’s *ρ* was used to assess the correlation between fixation time on specific aircraft and interventions in the analysis of errors in CD&R. A forward-stepwise (Wald) binomial logistic regression was used to determine the contribution of interventions and fixation times to CD&R. Significance was set at *α* = 0.05 for all analyses. Figures and analyses were produced with IBM SPSS v25 (Armonk, NY) and jamovi v2.3.

## 3 Results

### 3.1 MWL

#### 3.1.1 ISA

**Figure 3:**
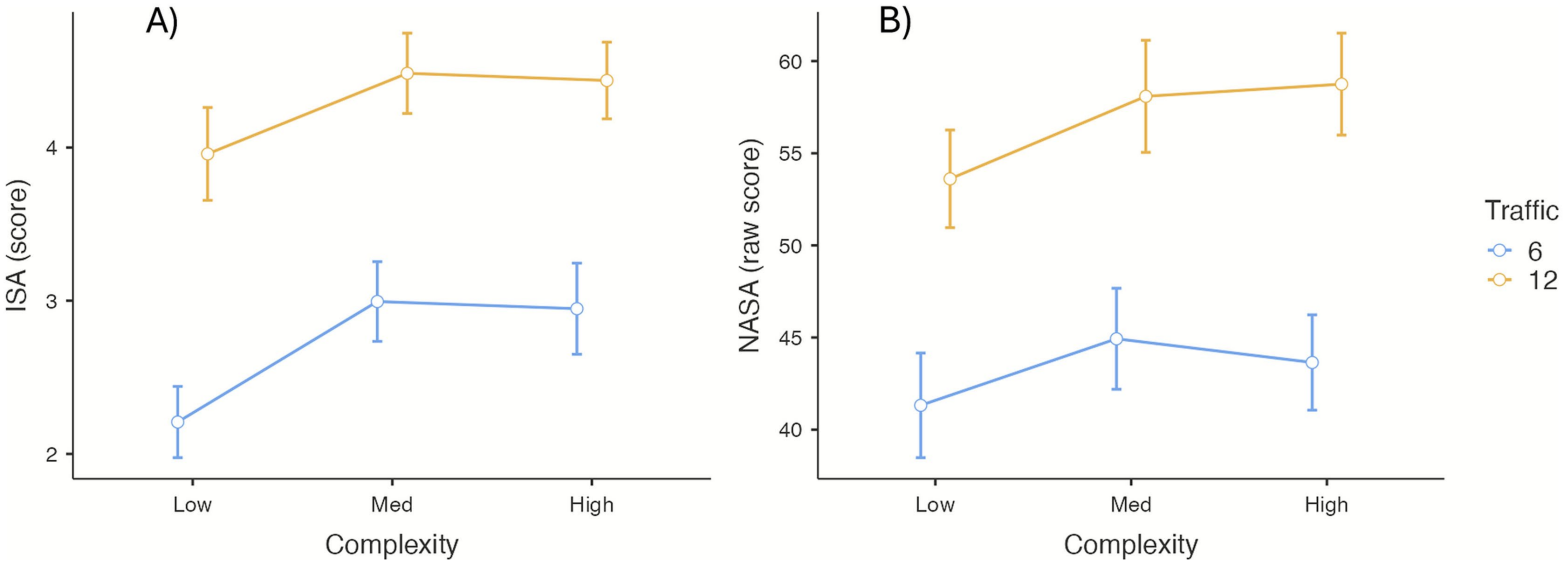
Mean values for ISA **(A)** and NASA **(B)** by traffic and complexity conditions. Error bars represent the standard error of the mean.

Repeated-measures ANOVA showed significant main effects of traffic, *F*_(1, 23)_ = 92.81, *p* < 0.001, *η_p_*^2^ = 0.80, and airspace complexity, *F*_(2, 46)_ = 7.48, *p* < 0.005, *η_p_*^2^ = 0.25; their interaction was not significant. ISA scores were higher under high-traffic conditions than under low-traffic conditions. Post-hoc comparisons indicated lower ISA estimates in low-complexity scenarios compared with medium- and high-complexity scenarios (Figure 3A).

#### 3.1.2 NASA

The NASA-TLX results displayed a similar pattern (Figure 3B). Significant main effects were observed for traffic, *F*_(1, 23)_ = 46.89, *p* < 0.001, **η_p_*^2^* = 0.67, and complexity, *F*_(2, 46)_ = 4.67, *p* < 0.05, *η_p_*^2^ = 0.17; the interaction was non-significant. Scores were higher in high-traffic scenarios, and post-hoc comparisons between complexity levels were significant only between low and medium complexity (*P_Bonf_* < 0.05).

### 3.2 Global metrics of visual behavior

#### 3.2.1 Pupil size

**Figure 4:**
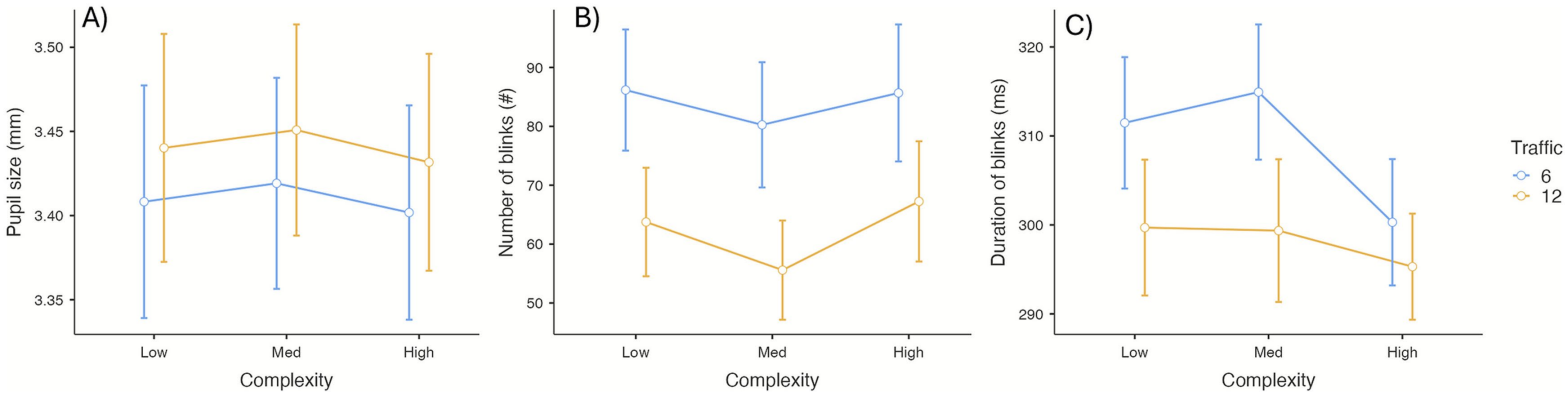
Mean values for pupil size **(A)**, number of blinks **(B)**, and blinks duration **(C)** by traffic and complexity conditions. Error bars represent the standard error of the mean.

Pupil diameter (Figure 4A) was larger in high-traffic scenarios (*M* = 3.44 ± 0.32 mm) than in low-traffic scenarios (*M* = 3.41 ± 0.32 mm), *F*_(1, 23)_ = 14.04, *p* < 0.001, *η_p_*^2^ = 0.38. Neither complexity, *F*_(2, 46)_ = 0.61, *p* = 0.550, *η_p_*^2^ = 0.03, nor the interaction, *F*_(2, 46)_ < 0.01, *p* = 0.996, *η_p_*^2^ < 0.01, reached significance.

#### 3.2.2 Number of blinks

Blink frequency (Figure 4B) decreased markedly under high traffic, *F*_(1, 23)_ = 47.24, *p* < 0.001, *η_p_*^2^ = 0.67. Complexity also produced a main effect, *F*_(2, 46)_ = 3.80, *p* < 0.05, *η_p_*^2^ = 0.14; the interaction was non-significant, *F*_(2, 46)_ = 0.50, *p* = 0.611, *η_p_*^2^ = 0.02. Post-hoc comparisons indicated fewer blinks in high- than in medium-complexity scenarios (*PBonf* < 0.05).

#### 3.2.3 Duration of blinks

Blink duration (Figure 4C) was shorter in high-traffic scenarios, *F*_(1, 23)_ = 5.60, *p* < 0.05, *η_p_*^2^ = 0.20. Complexity, *F*_(2, 46)_ = 2.25, *p* = 0.116, **η_p_*^2^* = 0.09, and the interaction, *F*_(2, 46)_ = 0.70, *p* = 0.501, *η_p_*^2^ = 0.03, were non-significant.

### 3.3 Static AOIs fixations

#### 3.3.1 Total fixation time

**Figure 5:**
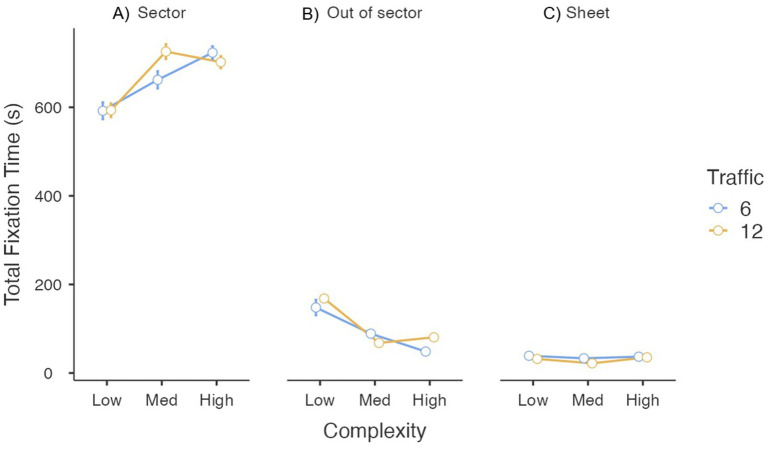
Total fixation time as a function of traffic and complexity conditions. Error bars represent the standard error of the mean.

Participants devoted 83% of their on-screen fixation time to the active sector (Figure 5A). Fixation time in this sector increased with scenario complexity, *F*_(1.63, 37.5)_ = 64.99, *p* < 0.001, *η_p_*^2^ = 0.74, but was unaffected by traffic, *F*_(1, 23)_ = 2.85, *p* = 0.105, *η_p_*^2^ = 0.11. A significant traffic × complexity interaction was observed, *F*_(2, 46)_ = 10.81, *p* < 0.001, *η_p_*^2^ = 0.32; simple-effect analyses showed that the difference between traffic levels was larger in the medium-complexity condition than in the low (*p* < 0.005) and high (*p* < 0.001) conditions.

Fixation time outside the active sector accounted for 13% of total fixation time (Figure 5B). No effect for traffic was found, *F*_(1, 23)_ = 2.81, *p* = 0.107, *η_p_*^2^ = 0.11, whereas it was confirmed a large main effect for complexity *F*_(1.22, 28.02)_ = 61.59, *p* < 0.001, *η_p_*^2^ = 0.73. Contrary to the active sector, the greater the complexity the fewer total time looking out of the active sector. The interaction was also significant, *F*_(1.65, 37.89)_ = 6.38, *p* < 0.01, *η_p_*^2^ = 0.22; the difference between traffic levels was smaller in the medium-complexity condition than in the low (*p* < 0.05) and high (*p* < 0.001) conditions.

Only 4% of fixation time was directed to the flight-plan sheet (Figure 5C). Viewing time was longer in six-flight scenarios, *F*_(1, 23)_ = 8.30, *p* < 0.01, *η_p_*^2^ = 0.27; complexity and the interaction were not significant (*p* > 0.10).

#### 3.3.2 Total number of fixations

**Figure 6:**
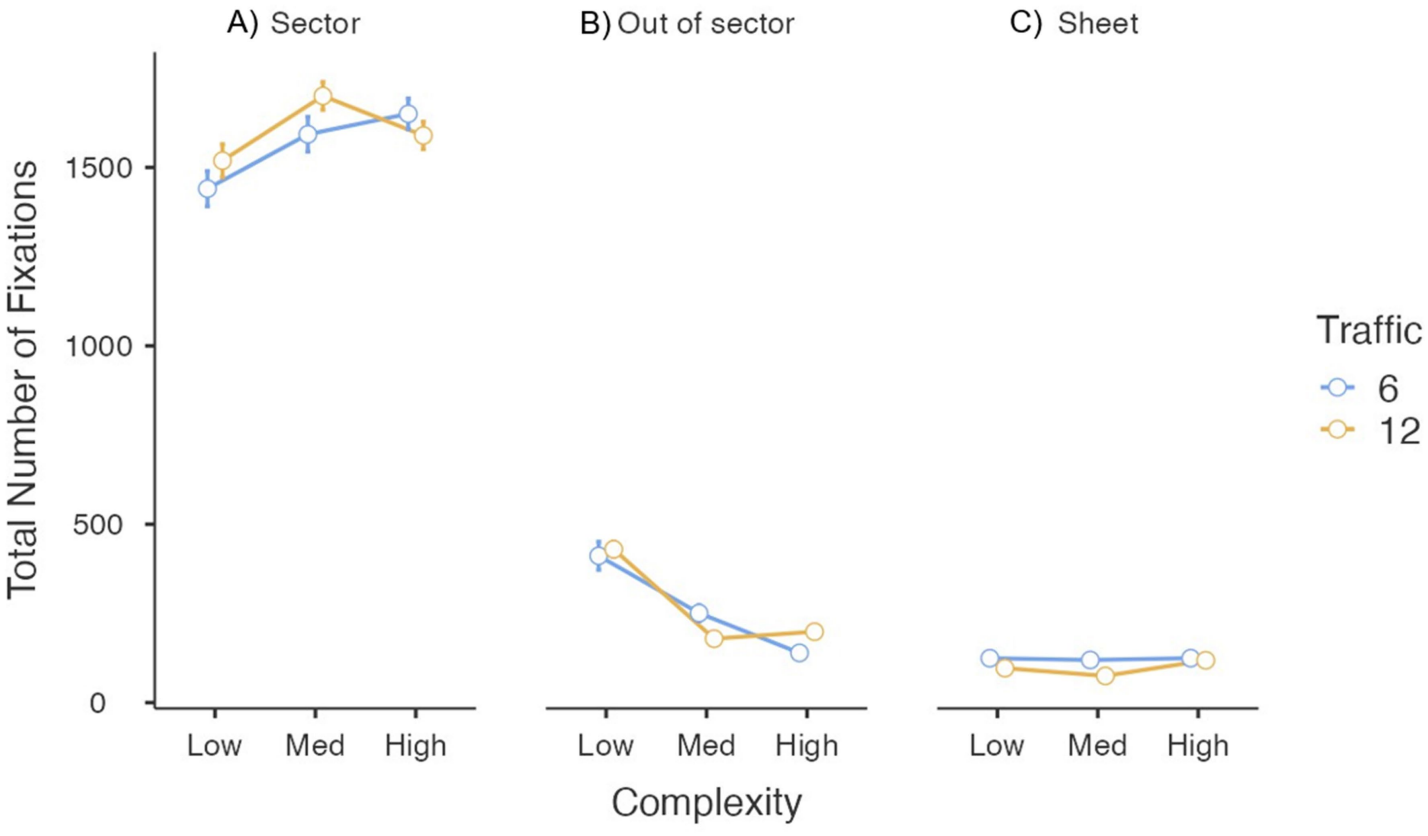
Total number of fixations as a function of traffic and complexity conditions. Error bars represent the standard error of the mean.

More than 80% of fixations fell within the active sector (Figure 6A). The number of fixations increased with complexity, *F*_(2, 46)_ = 16.94, *p* < 0.001, *η_p_*^2^ = 0.42., and there was a significant traffic × complexity interaction *F*_(2, 46)_ = 7.44, *p* < 0.005, *η_p_*^2^ = 0.24. Post-hoc tests showed more fixations in medium- and high-complexity scenarios than in low-complexity scenarios (all *PBonf* < 0.001). Differences between traffic levels appeared only in the medium-complexity condition. Traffic alone had no effect, *F*_(1, 23)_ = 1.48, *p* = 0.236, *η_p_*^2^ = 0.06.

Fixations outside the active sector represented 14% of total fixations (Figure 6B). Complexity again had a marked effect, *F*_(1.36, 31.31)_ = 89.43, *p* < 0.001, *η_p_*^2^ = 0.80, with fewer fixations as complexity increased. Traffic was non-significant, *F*_(1, 23)_ = 0.02, *p* = 0.900, *η_p_*^2^ < 0.01, but the interaction was significant, *F*_(1.75, 40.14)_ = 6.28, *p* < 0.01, *η_p_*^2^ = 0.21; differences between traffic levels were evident in the medium- and high-complexity conditions.

The flight-plan sheet attracted only 6% of fixations (Figure 6C). More fixations occurred during six-flight scenarios, *F*_(1, 23)_ = 14.30, *p* < 0.001, *η_p_*^2^ = 0.38; complexity, *F*_(2, 46)_ = 1.56, *p* = 0.222, *η_p_*^2^ = 0.06, and the interaction, *F*_(2, 46)_ = 1.96, *p* = 0.153, *η_p_*^2^ = 0.08, were non-significant.

### 3.4 Correlations between average MWL and global metrics of visual behavior

Spearman correlations across the six traffic × complexity conditions (Table 1) revealed a strong positive association between ISA and NASA-TLX scores (*p* = 0.017, 95% CI = 0.54–0.99). ISA correlated positively with pupil size (*p* = 0.033, 95% CI = 0.23, 0.99) and negatively with blink count (*p* = 0.017, 95% CI = −0.99, −0.54), indicating larger pupils and fewer blinks when self-reported MWL was higher.

**Table 1:** Spearman’s *ρ* correlation matrix between visual behavior measures and subjective measures of MWL.

	ISA	Pupil size	Num blinks	Dur blinks	Fix. time sector	Fix. time sheet
ISA	—	0.89*	−0.94*	−0.77	0.60	−0.83
NASA-TLX	0.94*	0.77	−0.83	−0.83	0.49	−0.66

*p < 0.05, **p < 0.01, ***p < 0.001, df = 4.

### 3.5 CD&R

**Figure 7:**
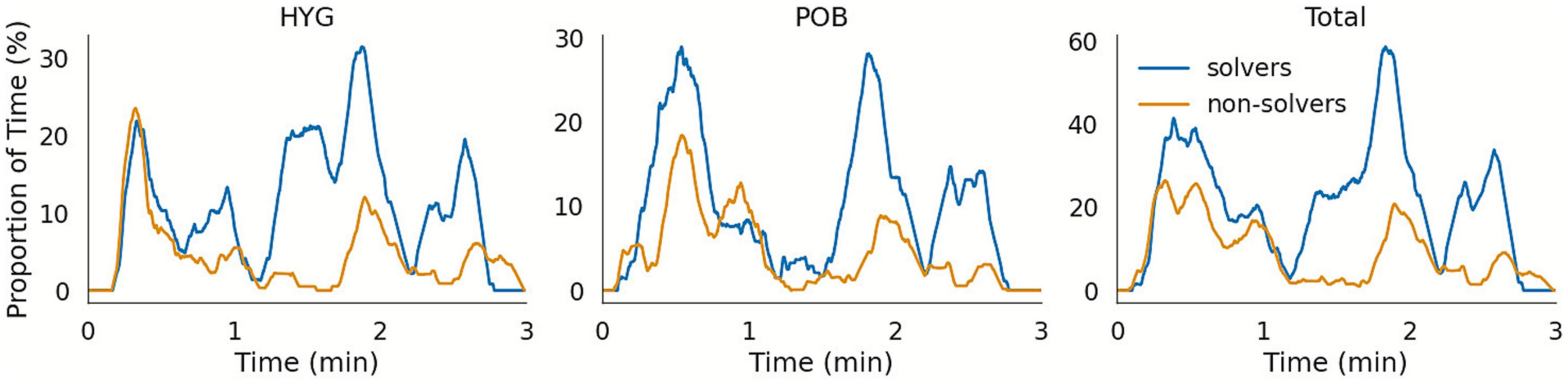
Instantaneous percentage of fixation time spent on aircraft involved in the Easy conflict in a 10 s sliding windows, for the first 3 min of the scenario (ending after the violation of the security constraints, programmed to happen after 140 s). Fixation times on POB456 (indicated as *POB* in the figure) and HYG532 (*HYG*) are presented in the left and center panels, and their sum (*Total*) in the right-most panel.

**Figure 8:**
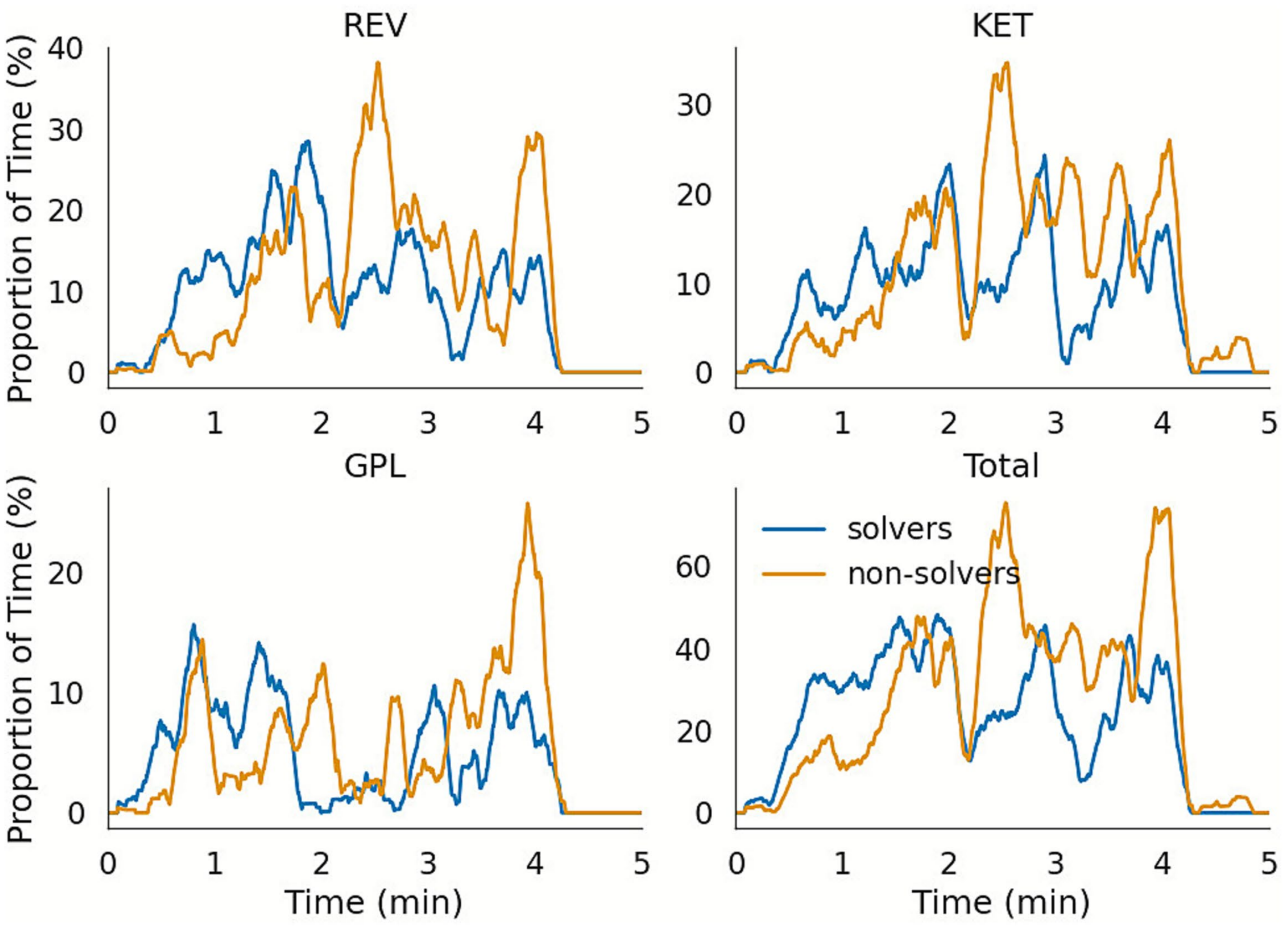
Instantaneous percentage of fixation time spent on aircraft involved in the Difficult conflict in a 10 s sliding windows, for the first 5 min of the scenario (ending after the violation of the security constraints, programmed to happen after 240 s). Fixation times on REV756 (indicated as *REV* in the figure) and KET456 (*KET*) are presented in the top row, whereas GPL751 (*GPL*) and the sum of the fixation times for the three aircraft (*Total*) are presented in the bottom row.

To investigate how gaze behavior relates to success or failure in CD&R, we first examined the temporal dynamics of fixations on the conflicting aircraft. Figures 7, 8 represent the percentage of time that participants fixated on each aircraft involved in the Easy and Difficult conflict scenarios respectively, using a 10-s moving window. These plots are shown separately for participants who successfully resolved the conflict (“solvers”) and those who did not (“non-solvers”), providing a direct comparison of information-gathering strategies across time. The resulting time courses reveal clear differences in gaze patterns: for the Easy conflict, solvers directed significantly more gaze time toward the conflicting aircraft (up to three times more) especially in the minute preceding the safety violation, while non-solvers failed to do so. In the Difficult conflict, both groups showed high levels of fixation to the relevant aircraft, with fixations reaching up to 80% of the time, particularly around the moment of conflict onset. These dynamic visualizations indicate that simplified metrics can yield meaningful insights into underlying cognitive processes in complex CD&R tasks.

#### 3.5.1 Easy conflict (HYG532-POB456)

Only 8 of 24 participants (33%) successfully resolved this conflict. Successful participants looked longer at the two conflicting aircraft, changed the altitude of HYG532 more often, and performed more total interventions (Table 2). Total fixation time correlated positively with the number of interventions (Table 3). Logistic regression identified the number of altitude interventions on HYG532 as the only significant predictor of success, *χ*^2^(1) = 17.99, *p* < 0.001, *R^2^MF* = 0.59, *VIF* = 1.

**Table 2:** Descriptive statistics and Mann–Whitney *U-* tests of the variables analyzed during the conflicts, split by successful intervention over the conflict.

Easy conflict: HYG532-POB456					Difficult conflict: REV756-KET456-GPL751				
	Solved		Unsolved			Solved		Unsolved	
	*n* = 8		*n* = 16			*n* = 13		*n* = 11	
Variables	*M*	*SD*	*M*	*SD*	Variables	*M*	*SD*	*M*	*SD*
Time accep. HYG (s)	11.13	11.89	14.44	23.35	Time in REV (s)	29.22	14.07	30.81	7.49
Time in HYG (s)**	18.29	6.25	8.08	6.13	Time in KET (s)	25.34	14.07	33.22	15.69
Time in POB (s)*	17.38	9.56	8.04	6.83	Time in GPL (s)	14.00	5.00	17.47	10.44
Total time (s)***	35.67	12.41	16.11	10.19	Total time (s)	68.56	27.83	81.50	19.55
Altit. HYG (#)***	0.75	0.46	0.00	0.00	Altit. REV (#)**	1.00	0.58	0.18	0.40
Altit. POB (#)	0.25	0.46	0.06	0.25	Altit. KET (#)	0.38	0.51	0.09	0.30
Speed HYG (#)	0.00	0.00	0.00	0.00	Altit. GPL (#)	0.54	0.52	0.27	0.47
Speed POB (#)	0.00	0.00	0.00	0.00	Speed REV (#)	0.08	0.28	0.09	0.30
Total interv. (#)***	1.00	0.53	0.06	0.25	Speed KET (#)	0.08	0.28	0.09	0.30
					Speed GPL (#)	0.08	0.28	0.00	0.00
					Total interv (#)**	2.15	0.80	0.73	0.79

*p < 0.05, **p < 0.01, ***p < 0.001.

**Table 3:** Spearman’s *ρ* correlation matrix between gaze variables (accumulated fixation times) and interventions for the Easy conflict.

	Time in HYG (s)	Time in POB (s)	Total time (s)
Time accept. HYG (s)	−0.37	−0.07	−0.25
Altitude HYG (#)	0.60**	0.48*	0.58**
Altitude POB (#)	0.05	0.41*	0.28
Total interventions (#)	0.46*	0.60**	0.61**

*p < 0.05, **p < 0.01, ***p < 0.001.

#### 3.5.2 Difficult conflict (REV756-KET456-GPL751)

Eleven participants (44%) resolved the second conflict. Mann–Whitney *U* tests showed that successful resolution was associated with more altitude interventions on REV756 and a higher total number of interventions (Table 2). There was no significant correlation between these variables and gaze dynamics (Table 3). The stepwise logistic model (*R*^2^*MF* = 0.64) indicated that the probability of success increased with the total number of interventions, *χ*^2^(1) = 19.27, *p* < 0.001, and decreased with longer overall fixation time, *χ*^2^(1) = 7.34, *p* = 0.007, *VIF* = 1.99 (see Table 4). Detailed regression outputs are provided in Supplementary Material 3 and a table with the details of the CD&R performance from all participants can be found in Supplementary Material 4.

**Table 4:** Spearman’s *ρ* correlation matrix between gaze variables (accumulated fixation times) and interventions for the Difficult conflict.

	Time in REV (s)	Time in KET(s)	Time in GPL(s)	Total time(s)
Altitude REV (#)	0.30	−0.20	−0.24	−0.06
Altitude KET (#)	−0.24	0.24	−0.04	−0.04
Altitude GPL (#)	−0.17	−0.02	0.42*	−0.02
Speed REV (#)	0.46*	0.30	0.33	0.48*
Speed KET (#)	0.13	0.22	0.20	0.22
Speed GPL (#)	0.32	0.05	0.05	0.14
Total interventions (#)	0.23	0.14	0.20	0.17

*p < 0.05, **p < 0.01, ***p < 0.001.

## 4 Discussion

The present study evaluated the visual behavior and the tactical interventions during simulated en route ATC tasks and related them to MWL and CD&R strategies. Using an ATC simulator, we systematically manipulated traffic load and airspace complexity factors known to modulate perceived MWL (Ibáñez-Gijón et al., 2023). Visual behavior was captured across multiple scales, ranging from broad indices such as pupil diameter, blink rate, and fixations to static scenario-level AOIs, to flight-specific fixation times obtained with dynamic AOIs in CD&R situations. The main findings are discussed below.

### 4.1 ISA and NASA-TLX

Consistent with previous work (e.g., Abdul Rahman et al., 2018), participants reported higher MWL both during the scenarios (ISA) and after completion (NASA-TLX) when traffic was heavy. Airspace complexity also affected subjective MWL, increasing across task workload levels, except between the medium- and high-complexity conditions. The absence of a significant difference may be due to local density effects because in the medium-complexity scenario, the same number of flights was concentrated along fewer routes, resulting in higher local traffic density which can elevate MWL (Loft et al., 2007). Our results therefore replicate those of Ibáñez-Gijón et al. (2023), evidencing the production of at least two clearly differentiated workload regimes (low and high MWL), and demonstrate that the eye-tracking equipment itself did not influence perceived MWL in our setup.

### 4.2 Pupil size and blinks

Mean pupil diameter (~3.4 mm) was smaller than the 5.7 mm reported by Bernhardt et al. (2019) and aligns with values observed under comparable luminance conditions (Pfleging et al., 2016). Pupil size increased with traffic load; although the absolute change was modest (~1%), the effect size was large (*η_p_*^2^ = 0.38), and pupil diameter correlated positively with ISA scores. Together, these findings reinforce the usefulness of pupillometry as an indicator of MWL.

Blink duration (295–315 ms) matched values for involuntary blinks in similar tasks (Sato et al., 2015). As traffic increased, participants blinked less often and for shorter periods. To our knowledge, this is the first evidence that higher traffic can shorten blink duration (cf. Meyer et al., 2022). This pattern diverges from the well-documented effects of fatigue, which typically lead to increased blink frequency and longer eye closure times (markers of cognitive disengagement or drowsiness observed in extended or monotonous tasks; Tautz and Tenoort, 2001; Vogt et al., 2006). This indicates that in our experiment blink behavior was not primarily driven by fatigue. Instead, the decrease in both blink rate and duration with increased traffic suggests an adaptive response to heightened perceptual and cognitive demands. This interpretation aligns with the perspective offered by Fairclough et al. (2005), who proposed that task engagement controls blink regulation before the effect of fatigue dominates (after 25 min in their study). Under high task workload, the suppression and shortening of blinks may serve to maximize visual intake and prevent the loss of critical information.

### 4.3 Fixations per AOI

Participants devoted more than 80% of fixation time and count to the active sector. As scenario complexity rose, gaze was increasingly concentrated within the sector, implying that a denser or more conflict-prone airspace narrows attentional focus (Martin et al., 2011). Conversely, when more flights had to be managed, participants consulted flight-plan strips less often and with shorter fixations, indicating a strategic shift toward conflict avoidance rather than adjustment of potential discrepancies before hand-off.

### 4.4 CD&R

Two potential conflicts with varying difficulty were analyzed. In the Easy conflict, changes in altitude of the non-standard-route flight (HYG532) were decisive to solve it: Six of the eight successful resolutions included an altitude change, whereas none of the unsuccessful attempts did so. Fixation time on HYG532 correlated with both altitude interventions and total interventions, suggesting that longer gaze reflects earlier conflict awareness and, consequently, more effective control actions (Martin et al., 2011). Failures therefore appear linked to insufficient monitoring.

The Difficult conflict involved three flights, one climbing along a non-standard route. Successful participants executed more interventions, especially altitude changes. Unlike the Easy conflict, in the Difficult conflict longer fixation time did not predict success. Here, most participants detected the conflict but some failed to anticipate the inter-dependencies among flights, indicating shortcomings in planning rather than monitoring.

Taken together, these findings support our hypothesis that the type of error in CD&R in our experiment is influenced by the difficulty of the conflict: Misses in the simpler dyadic conflicts primarily reflect lapses in the detection phase, whereas failures in the more complex triadic conflicts arise from shortcomings in the planning phase after conflicts have been identified. This pattern aligns with the Conflict Life-Cycle framework (Meyer et al., 2022; Nordman et al., 2023), which links gaze and decision behavior to phase-specific cognitive demands. Our results thus reinforce evidence that different Life-Cycle phases entail distinct cognitive mechanisms and error vulnerabilities. To refine this interpretation, complementary approaches such as retrospective verbal protocols (Nordman et al., 2023; Palma Fraga et al., 2021) could help further disentangle phase-specific processes, especially those underlying planning failures.

### 4.5 Limitations

Our primary limitation is the use of novice participants who were trained specifically for this study. Recruiting a sufficiently large sample (*N* = 24) made it impracticable to involve professional ATCOs. Only three previous eye-tracking studies (Martin et al., 2011; Tautz and Tenoort, 2001; Vogt et al., 2006) have included comparable or larger professional samples. Consequently, absolute MWL estimates and visual behavior metrics may exaggerate the values that experienced ATCOs would exhibit. Prior research has shown that professional ATCOs adopt efficient and goal-directed gaze strategies, with systematic visual scanning patterns and large individual differences in the specific details of the strategy performed (McClung and Kang, 2016; Palma Fraga et al., 2021). Novices, by contrast, may engage in more reactive, exhaustive or disorganized search behaviors, leading to higher fixation rates and potentially elevated MWL ratings under comparable task conditions. As such, our MWL estimates may overstate the cognitive demands that would be reported by experienced ATCOs in the same scenarios, while visual metrics may reflect learning-related effort rather than operational norms. Nonetheless, the statistical power of our design allows us to relate changes in MWL and visual search robustly and to offer reference values compatible with analogous non-ATC tasks. We also believe that the findings provide a useful benchmark for how attentional strategies emerge and adapt in early-stage ATC performance, setting the stage for future expert-novice comparisons.

Another limitation of the present study is the uncertainty surrounding the exact moment participants detected a conflict, because detection was inferred from gaze behavior and intervention timing. While this approach provided clear classifications in most cases within our novice sample, it may be less reliable in more complex or operationally realistic contexts, particularly with expert participants. Future studies would benefit from the inclusion of think-aloud protocols (Palma Fraga et al., 2021) to more precisely assess situational awareness and support further interpretation of gaze patterns.

A third potential limitation concerns the sampling frequency of the eye-tracking device employed in the study, which operated at 30 Hz. While this rate is sufficient for capturing general gaze behavior and fixation patterns, it may limit the precise detection of brief saccades or micro-events that occur below the temporal sampling threshold. This can affect the timing and resolution of event-level gaze metrics, particularly in fast-paced visual tasks. However, it is important to note that our analyses were based on aggregated fixation durations and spatial distribution metrics rather than the specific timing of micro events. These higher-level measures are more robust to the limitations imposed by lower sampling frequencies, as they rely on accumulated gaze behavior over extended time windows.

## 5 Conclusion

The present study advances predictive modeling of ATCO MWL and error in CD&R. We find that pupil dilation, blink behavior, and global fixation patterns are reliable MWL markers, and that short-timescale gaze–action dynamics around scripted conflicts help explain why resolutions fail. Consistent with our hypotheses, errors in two-aircraft conflicts primarily stemmed from undetected encounters, whereas errors in three-aircraft conflicts reflected planning breakdown despite successful detection. These dissociable mechanisms suggest two complementary supports: alerting when a conflict is unnoticed and recommending resolution options when planning stalls. Because the indicators derive from non-invasive eye-tracking, these advances indicate potential for eventual integration into operational workstations. Future work with professional ATCOs and broader conflict geometries is needed to refine these findings and define operational thresholds.

## Data Availability

The raw data supporting the conclusions of this article will be made available by the authors, without undue reservation.
