# Supplementary material for: Eye-tracking metrics for estimating workload and characterizing errors in conflict detection and resolution during simulated en route air-traffic control
Source: Front Psychol. 2025 Dec 10;16:1644721. doi: 10.3389/fpsyg.2025.1644721 (PMC12729096; doi:10.3389/fpsyg.2025.1644721)
Supplement: Supplementary file 1 [file Supplementary_file_1.docx]

Supplementary Material **1**

**Adjustment of the participant’s distance from the eye-tracker**

We analyzed the distance from the participant’s eyes to eye-tracker as a measure of methodological validity. The average value of 58.8 ± 2.3 cm kept within the Tobii recommendations for an optimal eye capture (Tobi Studio V.3.4.8). Moreover, it was observed that participants moved slightly closer to the screen (0.8 cm) when facing high traffic scenarios (12 aircrafts), F_(1,23)_ = 9.97, p < .005, η_p_^2^ = .30. Neither complexity as a main factor, F_(1,46)_ = 2.20, p = .122, η_p_^2^ = .09, nor the interaction, F_(1,46)_ = 1.83, p =.171, η_p_^2^ = .007, reached significance.


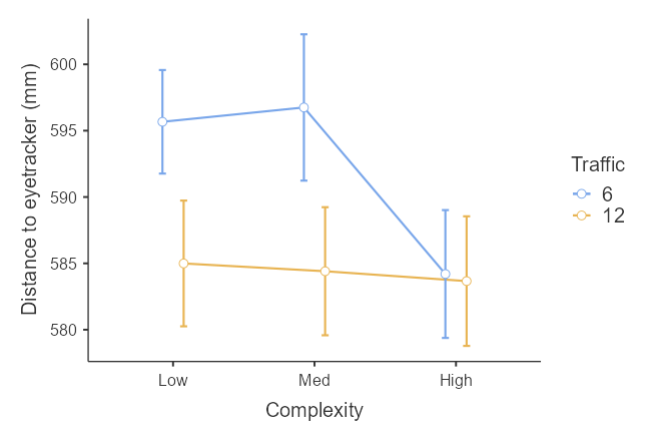


**Supplementary Figure 1.** Distance to the eye-tracker as a function of traffic and complexity conditions. Error bars represent the standard error of the mean.
