# Supplementary material for: Eye-tracking metrics for estimating workload and characterizing errors in conflict detection and resolution during simulated en route air-traffic control
Source: Front Psychol. 2025 Dec 10;16:1644721. doi: 10.3389/fpsyg.2025.1644721 (PMC12729096; doi:10.3389/fpsyg.2025.1644721)

Supplementary Material 2

**Screenshots per minute of the 12-high scenario containing the two conflicts**

**
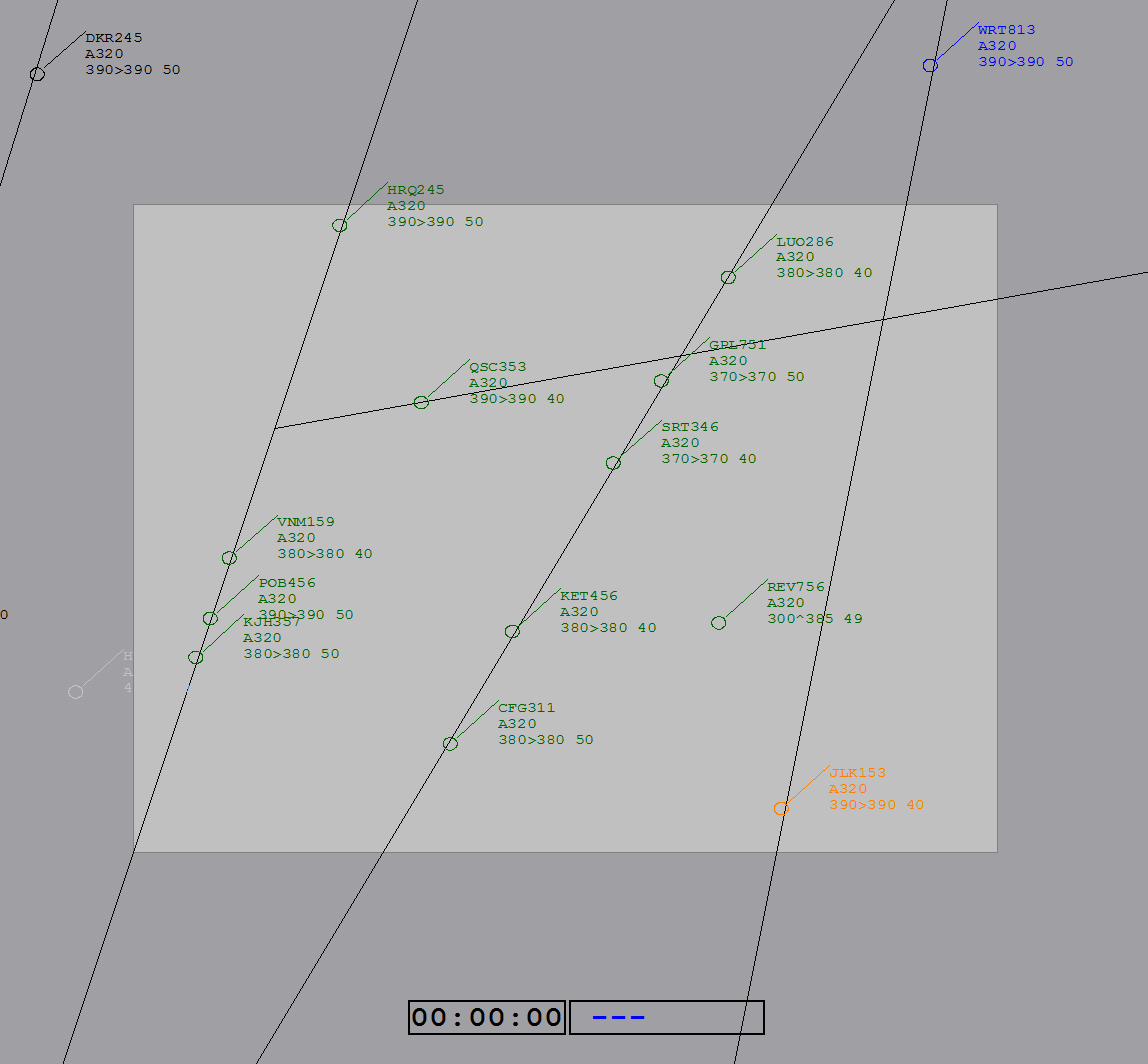
**


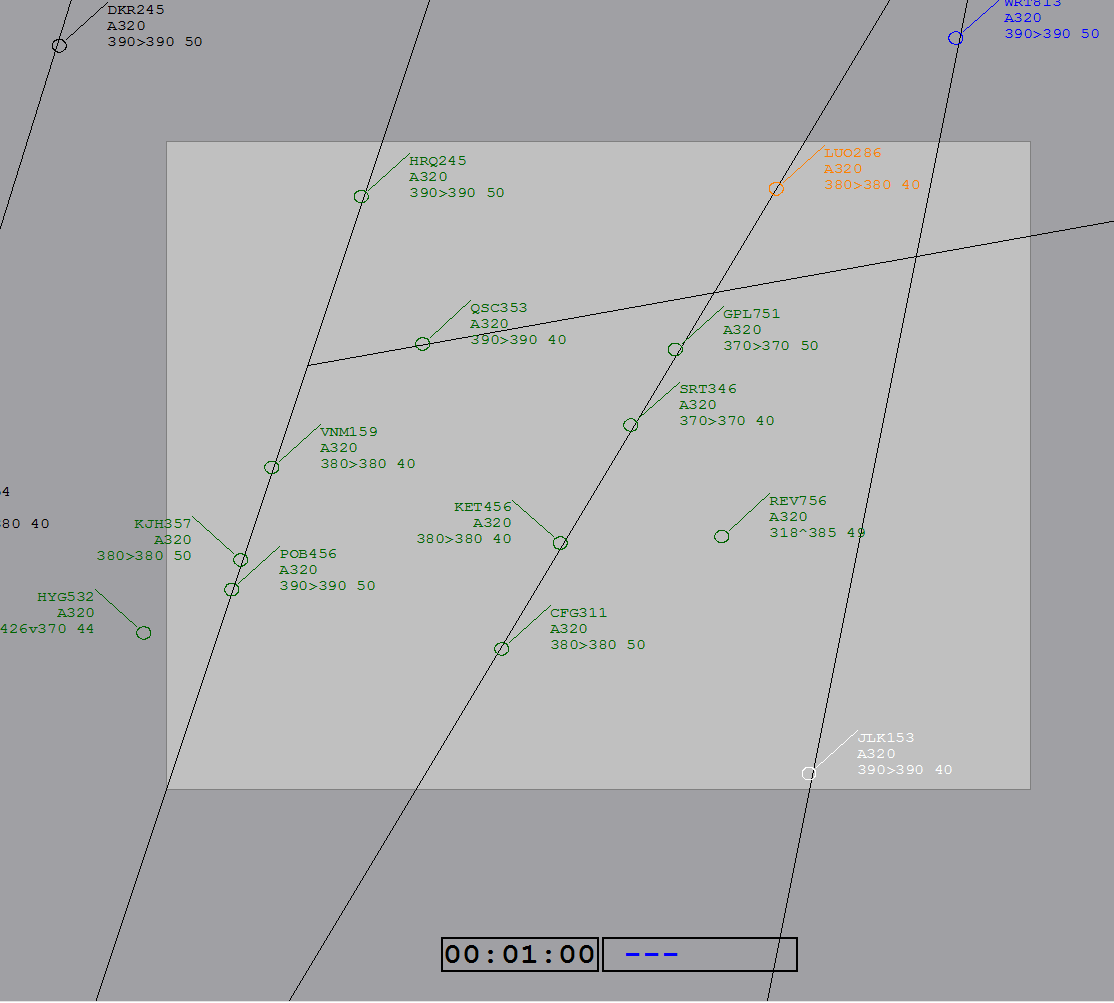


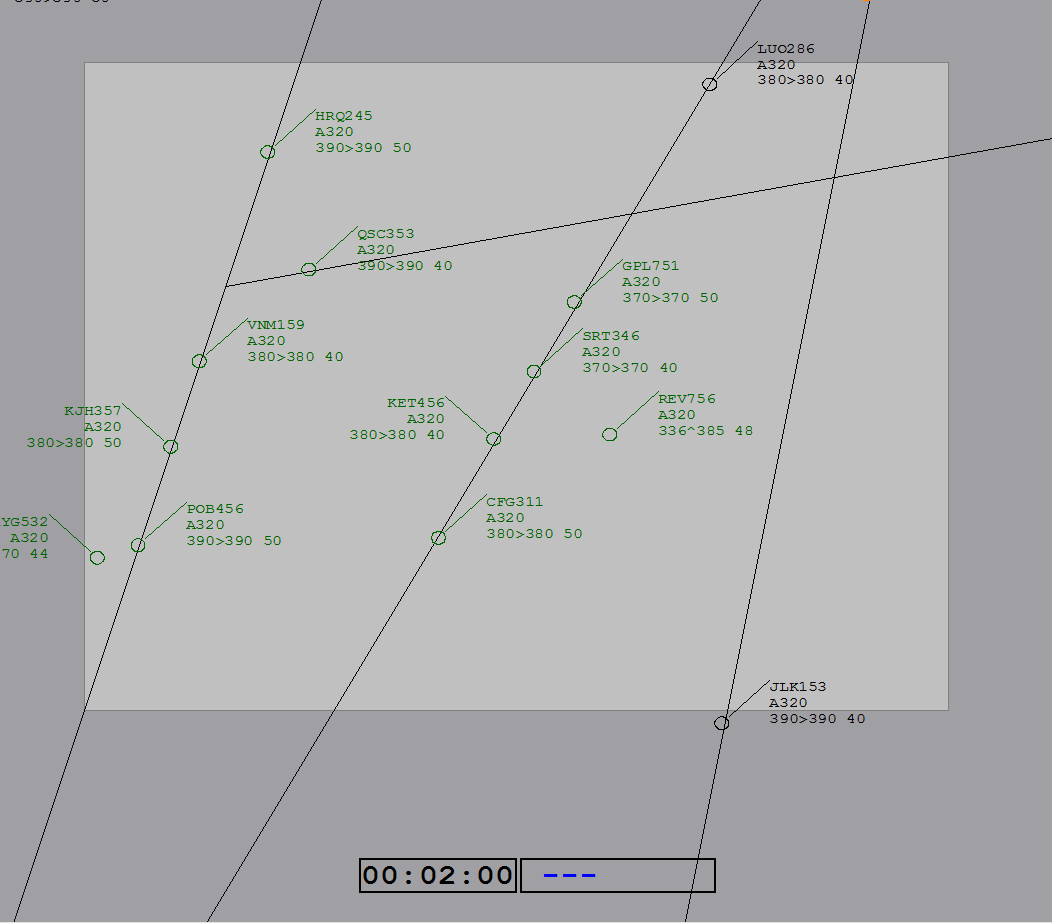

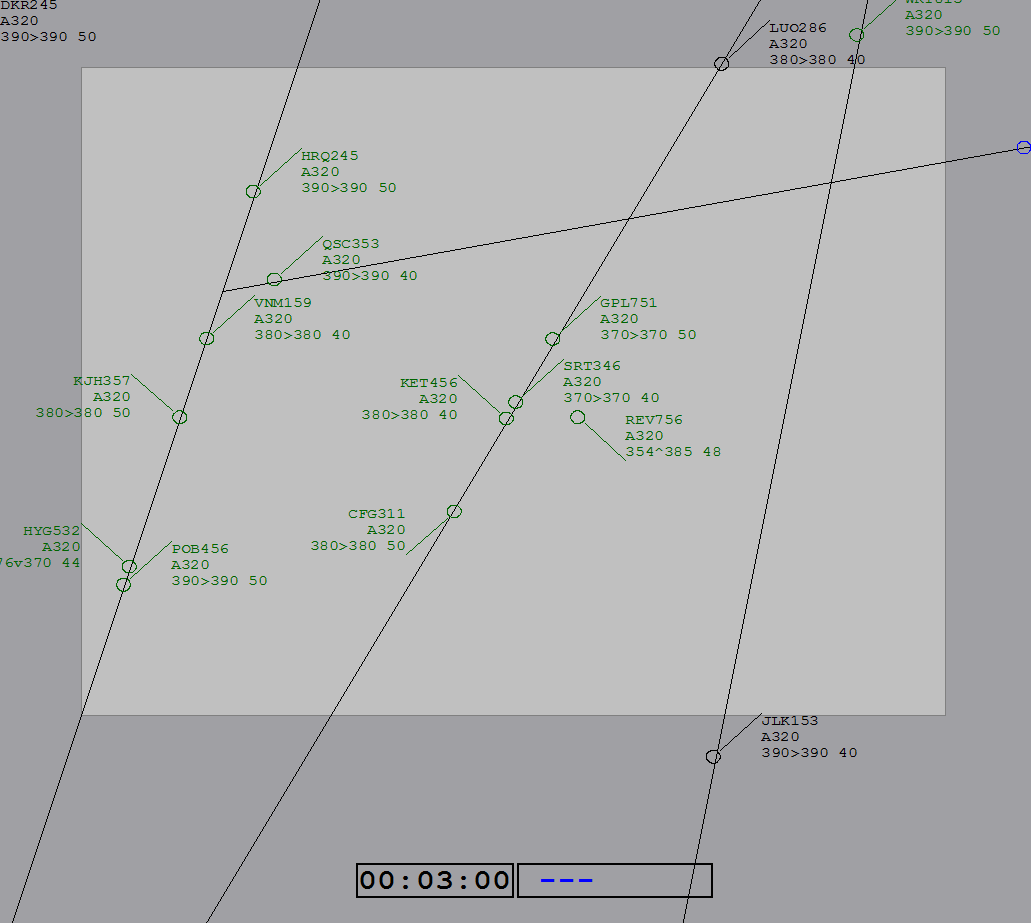


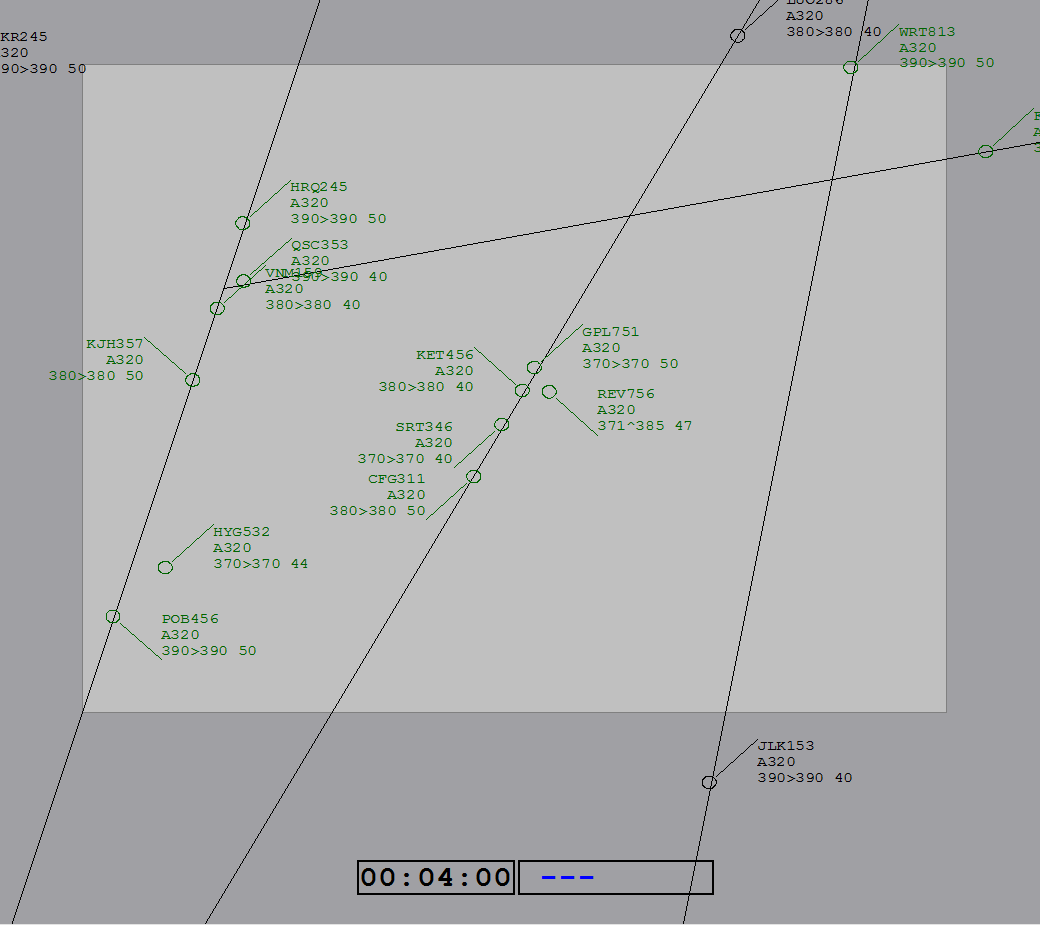

Supplement: Supplementary file 2 [file Supplementary_file_2.docx]
