# Supplementary material for: Eye-tracking metrics for estimating workload and characterizing errors in conflict detection and resolution during simulated en route air-traffic control
Source: Front Psychol. 2025 Dec 10;16:1644721. doi: 10.3389/fpsyg.2025.1644721 (PMC12729096; doi:10.3389/fpsyg.2025.1644721)
Supplement: Supplementary file 3 [file Supplementary_file_3.docx]

Supplementary Material 3

**Complementary information of binomial logistic regression models on conflict solving**

|  |  | Conflict | | | | | | |
| --- | --- | --- | --- | --- | --- | --- | --- | --- |
|  |  | First: HYG532-POB456 | | |  | Second: REV756-KET456-GPL751 | | |
|  |  | *B* | *SE* | *p* | *g* | *B* | *SE* | *p* |
| Intercept |  | -2.08 | .75 | .006 |  | 1.41 | 2.44 | .564 |
| Altitude HYG532 |  | 21.65 | 4390.31 | .996 |  |  |  |  |
| Total interventions |  |  |  |  |  | 3.27 | 1.29 | .011 |
| Total time of fixation |  |  |  |  |  | -.08 | .04 | .051 |
| *R^2^_McF_* |  | .59 |  |  |  | .64 |  |  |
| *p* |  | < .001 |  |  |  | < .001 |  |  |
| VIF |  | 1.00 |  |  |  | 1.99 |  |  |

**Supplementary Table 1.**


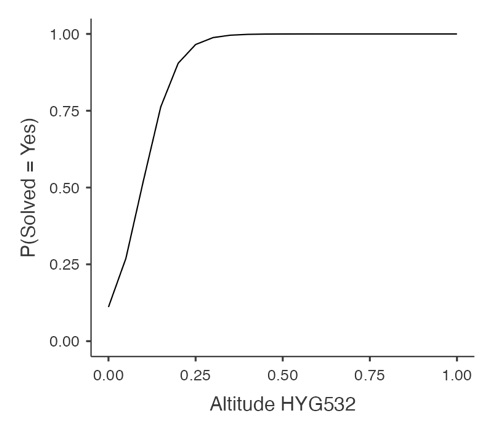

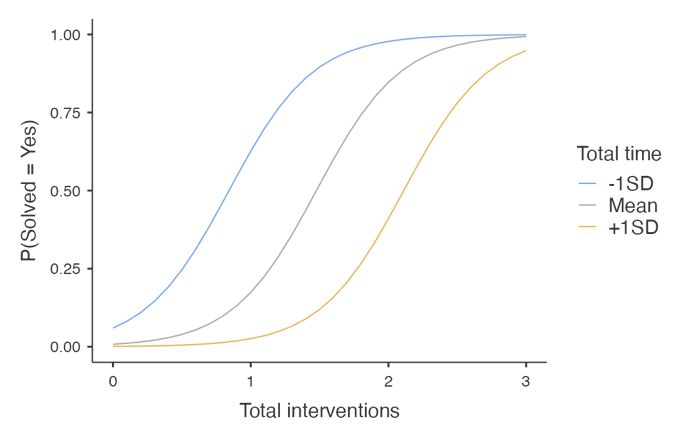


**Supplementary Figure 1.** Probability of solved conflict as a function of variables included in the model. Left panel for first conflict and right panel for second conflict.
